# Lipid Raft-Dependent FcεRI Ubiquitination Regulates Receptor Endocytosis through the Action of Ubiquitin Binding Adaptors

**DOI:** 10.1371/journal.pone.0005604

**Published:** 2009-05-19

**Authors:** Rosa Molfetta, Francesca Gasparrini, Giovanna Peruzzi, Laura Vian, Mario Piccoli, Luigi Frati, Angela Santoni, Rossella Paolini

**Affiliations:** Department of Experimental Medicine, Institute Pasteur-Fondazione Cenci Bolognetti, Sapienza University, Rome, Italy; University of Geveva, Switzerland

## Abstract

The best characterized role for ubiquitination of membrane receptors is to negatively regulate signaling by targeting receptors for lysosomal degradation. The high affinity receptor for IgE (FcεRI) expressed on mast cells and basophils is rapidly ubiquitinated upon antigen stimulation. However, the nature and the role of this covalent modification are still largelly unknown. Here, we show that FcεRI subunits are preferentially ubiquitinated at multiple sites upon stimulation, and provide evidence for a role of ubiquitin as an internalization signal: under conditions of impaired receptor ubiquitination a decrease of receptor entry is observed by FACS analysis and fluorescence microscopy. We also used biochemical approaches combined with fluorescence microscopy, to demonstrate that receptor endocytosis requires the integrity of specific membrane domains, namely lipid rafts. Additionally, by RNA interference we demonstrate the involvement of ubiquitin-binding endocytic adaptors in FcεRI internalization and sorting. Notably, the triple depletion of Eps15, Eps15R and Epsin1 negatively affects the early steps of Ag-induced receptor endocytosis, whereas Hrs depletion retains ubiquitinated receptors into early endosomes and partially prevents their sorting into lysosomes for degradation. Our results are compatible with a scenario in which the accumulation of engaged receptor subunits into lipid rafts is required for receptor ubiquitination, a prerequisite for efficient receptor internalization, sorting and delivery to a lysosomal compartment.

## Introduction

Ubiquitination is a post-translational reversible modification whereby a small and highly conserved peptide, ubiquitin (Ub), is covalently bound to acceptor proteins. This protein modification regulates several cellular functions including protein degradation, endocytosis of cell surface receptors, DNA damage repair, and virus budding [1]–[3].

Notably, whereas Lys 48-linked Ub chains target proteins for proteasomal degradation [1], conjugation of a single Ub moiety to one (monoubiquitination) or more (multiubiquitination) Lys residues of the substrate protein has been proposed to be sufficient as internalization and endocytic sorting signal in yeast [2], [4]. In mammalian cells, although a role in controlling the sorting of growth factor receptors to degradative compartments has been extensively documented [5]–[9], the requirement for Ub signal at the initial step of receptor internalization has remained more elusive [7], [8].

In the past years we have concentrated our interest in the role of Ag-induced ubiquitination of immune receptors focusing on the FcεRI, constitutively expressed on the membrane of mast cells and basophils.

FcεRI is composed by an IgE-binding α-chain, and β and γ subunits both able to transduce signals via the paired tyrosine residues located in their cytoplasmic motifs termed immunoreceptor tyrosine-based activation motifs (ITAMs).

It is generally accepted that upon FcεRI cross-linking, the β chain-associated Src family protein tyrosine kinase Lyn becomes activated, and phosphorylates the β and γ-chain ITAMs allowing the recruitment and activation of the cytoplasmic Syk tyrosine kinase.

This event is required for all known FcεRI-mediated mast cell responses, including the release of preformed and newly synthesized allergic mediators [10]–[13].

We have previously demonstrated that upon Ag stimulation FcεRI β and γ subunits are also ubiquitinated through the combined enzymatic activities of the tyrosine kinase Syk and the Ub ligase c-Cbl [14], [15]. Although our results suggest a role for this modification in the termination of cell activation, the role of receptor ubiquitination in the initial step of endocytosis and in the trafficking of engaged FcεRI complexes has not been investigated.

A major pathway of FcεRI internalization is clathrin-dependent endocytosis, whereby the receptor is removed from the cell surface via clathrin-coated pits and then routed to endocytic compartments for lysosomal degradation [16]–[18]. However, the existence of an alternative clathrin-independent and lipid raft-dependent endocytic pathway has also been recently envisaged [19], [20].

Lipid rafts are specialized regions of the plasma membrane enriched in cholesterol and glycosphingolipid that form ordered but dynamic structures floating in the less ordered surrounding membrane [21]. The role of lipid rafts in signaling is extensively documented, as they function as physical platforms capable to assemble the signal transduction machinery [21]–[23]. Upon engagement of FcεRI membrane rafts coalesce into larger and more stable structures where engaged receptors are concentrated and can more easily interact with signaling molecules, such as the active form of Src family kinases [24]–[28].

In the case of mast cells, lipid rafts play also a role in controlling the intensity and duration of FcεRI-induced signal in that the impairment of raft integrity enhances degranulation and cytokine release [26], [29]. Moreover, lipid rafts are competent for recruitment of negative regulators of signal transduction, such as the Ub ligase c-Cbl [30].

To gain insight in this issue, we have investigated the relationship between lipid rafts and FcεRI ubiquitination and their role in regulating receptor internalization and sorting.

We show that engaged FcεRI subunits are multiubiquitinated into lipid rafts and provide evidence that this modification controls receptor internalization and sorting along the endocytic compartments through the interaction with Ub-binding adaptors.

## Results

### FcεRI ubiquitination regulates Ag-induced receptor endocytosis

Multiubiquitination of membrane receptors has become recognized as an important signal for their internalization and transport along the endocytic machinery [2], [4]–[6], [8], [9].

In the case of FcεRI, we have previously reported the presence of several Ag-induced ubiquitinated β and γ forms (at least 3 species for β, and 6 species for γ) [14], but the nature of Ub modification remains unclear.

**Figure 1:**
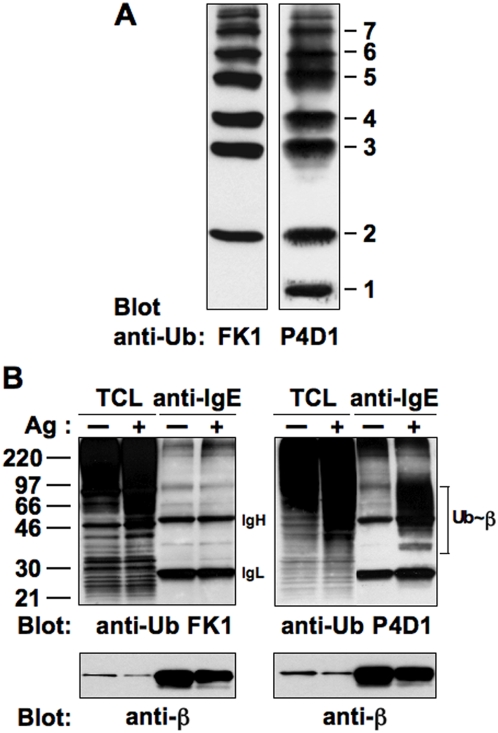
FcεRI is preferentially monoubiquitinated at multiple sites upon Ag binding. (A) The ability of P4D1 and FK1 anti-Ub mAbs to recognize a single Ub molecule or a poly-Ub chain, respectively, was tested by Western blotting using an Ub ladder. (B) RBL-2H3 cells (4×10^7^/sample) were sensitized with anti-DNP IgE, and stimulated (+) or not (−) with DNP-HSA (Ag) for 1 min at 37°C. Total cell lysates (TCL) and anti-IgE immunoprecipitates were resolved by SDS-PAGE and immunoblotted with the indicated Abs.

In order to discriminate between poly- or multiubiquitinated induced forms, we used commercially available anti-Ub mAbs with different specificities. As revealed by the immunoblot performed on a mixture of Ub monomers and polymers, the FK1 mAb recognized only poly-Ub chains [31], whereas the P4D1 recognized Ub monomers as efficiently as poly-Ub chains (Figure 1A).

RBL-2H3 cells were incubated overnight with anti-DNP IgE mAb and stimulated (or not) with the multivalent Ag DNP-HSA for 1 min at 37°C before receptor immunoprecipitation and Western blotting with anti-Ub mAbs (Figure 1B). FK1, although efficiently recognized polyubiquitinated proteins from total cell lysates, failed to detect modified receptor forms, whereas P4D1 was able to detect Ag-induced ubiquitinated forms of FcεRI β chain. A similar reactivity (not shown) was also observed by using FK2, another anti-Ub mAb recognizing both mono- and polyubiquitinated proteins [31].

Similar results were obtained for FcεRI γ chain (data not shown).

These finding suggest that upon Ag stimulation FcεRI undergoes multiple monoubiquitination but not efficient polyubiquitination.

We further decided to investigate whether FcεRI ubiquitination may control receptor endocytosis by evaluating the initial rate of Ag-induced receptor down-modulation and internalization under conditions of impaired ubiquitination.

As a first attempt, we used a recently described compound belonging to the family of pyrazones, namely UBE1-41, that can affect the initial step of the ubiquitination process by the covalent modification of the active site Cys of the Ub-activating enzyme E1 [32].

**Figure 2:**
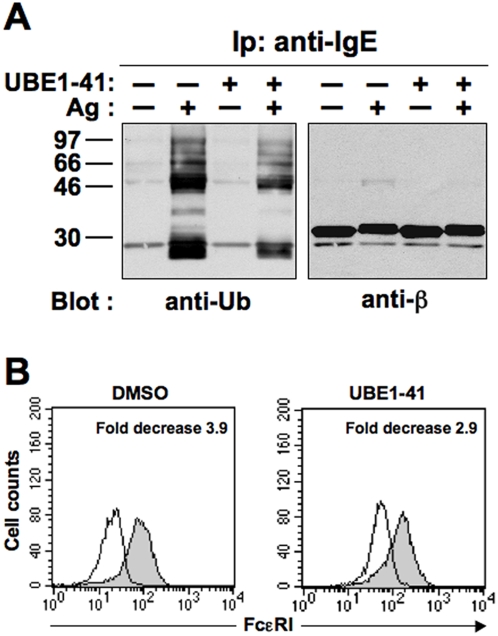
The E1 inhibitor UBE1-41 impairs Ag-induced FcεRI ubiquitination and internalization. (A) Sensitized RBL-2H3 cells (3×10^7^/sample) were pretreated with 50 µM UBE1-41, and then stimulated (+) or not (−) with Ag for 1 min at 37°C. Anti-IgE immunoprecipitates were resolved by SDS-PAGE, and immunoblotted with the indicated Abs. (B) Cells treated as in *A* were stimulated with Ag for 30 min and the FcεRI surface expression was measured by FACS analysis performed after the addition of a FITC-conjugated anti-IgE mAb. Fold decrease was calculated as ratio of the mean fluorescence intensity of unstimulated (closed histograms) vs stimulated samples (open histograms).

Cells preloaded with IgE were pretreated with vehicle alone (DMSO) or with 50 µM UBE1-41 before Ag stimulation (Figure 2). As a consequence of UBE1-41 treatment, Tyr phosphorylation of engaged FcεRI β subunit was increased in intensity (data not shown), as previously reported for ligand-induced EGFR phosphorylation [32]. Furthermore, consistent with its predicted activity, the Ag-induced ubiquitination of FcεRI β chain was partially inhibited (Figure 2A). Similar results were obtained for FcεRI γ chain (data not shown).

A concomitant reduction of Ag-induced receptor down-modulation was observed by FACS analysis (Figure 2B), suggesting the involvement of FcεRI ubiquitination in regulating receptor surface expression upon stimulation.

To further evaluate the effect of such inhibitor on receptor internalization, we performed fluorescence microscopic analysis. However, upon drug pretreatment we noticed an increase of autofluorescence likely due to the ring structure of the compound. To overcome this problem, we used different combinations of exciting and emission wavelenghts but the interference observed made unintelligible this set of experiments.

Thus, as a second attempt we employed a Syk-negative variant of RBL-2H3 cells (Syk^−^) in which the absence of active Syk dramatically reduces Ag-induced FcεRI ubiquitination [15], and cells obtained by stable transfection of the Syk^−^ cell line with WT (Syk^+^) or a kinase-dead mutant form of rat Syk (Syk KI) [33].

To compare the degree of receptor down-modulation in Syk^+^ versus Syk deficient cells, each cell line was sensitized with anti-DNP IgE mAb and stimulated (or not) with Ag for different lengths of time. Changes in cell surface FcεRI expression were analyzed by FACS analysis and fluorescence microscopy.

**Figure 3:**
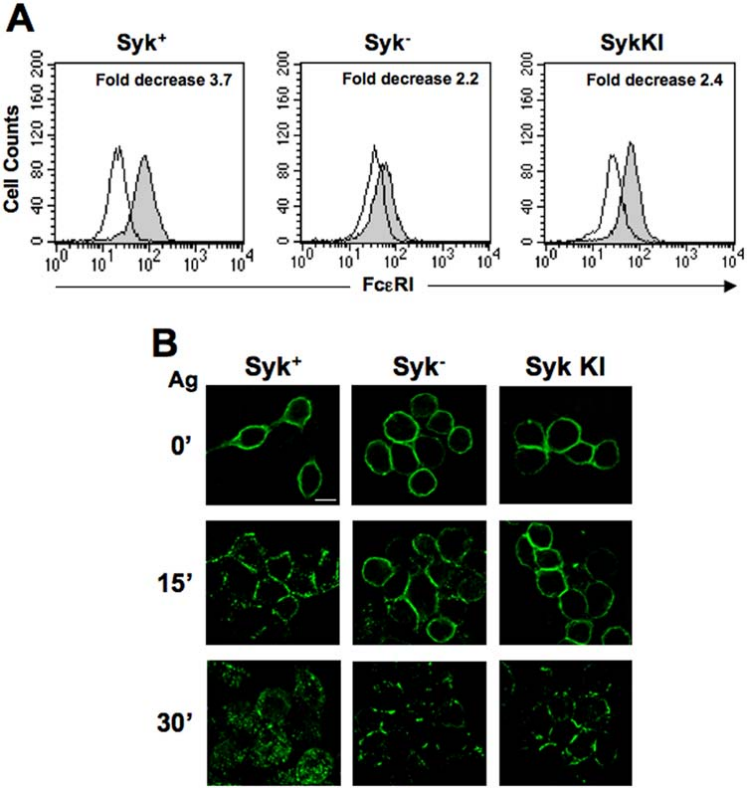
Syk regulates FcεRI endocytosis. (A) A Syk-negative variant of the RBL-2H3 cells (Syk^−^) and clones obtained by stable transfection of this cell line with WT Syk (Syk^+^) or a Syk kinase-dead mutant (KI) were stimulated with Ag for 30 min. FcεRI expression of unstimulated (closed histograms) and stimulated (open histograms) cells was evaluated by FACS analysis. Fold decrease was calculated as in Figure 2B. (B) Cells (120×10^3^) were grown for 12 h on round glass coverslips in the presence of anti-DNP IgE (0.3 µg), and stimulated with Ag for the indicated lenghts of time. IgE-FcεRI complexes were visualized with a FITC-conjugated anti-IgE mAb, and fluorescence images, shown as a single optical section, obtained using an ApoTome microscope. Scale bar indicates 10 µm. Results are representative of three independent experiments.

An attenuation of receptor down-modulation was observed upon 30 min of stimulation in Syk^−^ or Syk KI cells (Figure 3A), suggesting a role for active Syk in regulating FcεRI surface expression.

Upon 15 min of incubation at 37°C, microscopic analysis revealed a receptor redistribution in clustered zones only on the plasma membrane of WT Syk expressing cells (Figure 3B). After 30 min of stimulation, the receptors were concentrated in intracellular dots in the Syk^+^ cells, whereas they were still localized in dashed zone on the membrane of the Syk^−^ and Syk KI cells. These differences are statistically significant when quantified by correlation analysis of multiple cells (data not shown).

We have previously demonstrated the requirement of Syk kinase activity in Cbl-mediated receptor ubiquitination [15]; thus it is likely that Syk may also affect receptor internalization and trafficking by controlling receptor ubiquitination.

All together our results strongly suggest a role for receptor multiubiquitination in controlling the Ag-induced FcεRI internalization process.

### The integrity of lipid rafts is required for Ag-induced FcεRI endocytosis

FcεRI has been reported to translocate into lipid rafts upon Ag stimulation [24]–[26], [28], and the implication of a lipid raft environment in regulating the Ag-induced FcεRI ubiquitination has also been envisaged [30].

To further investigate the relationship between lipid rafts and Ag-induced FcεRI ubiquitination, we initially analyzed whether ubiquitinated receptor subunits are preferentially located into lipid rafts.

**Figure 4:**
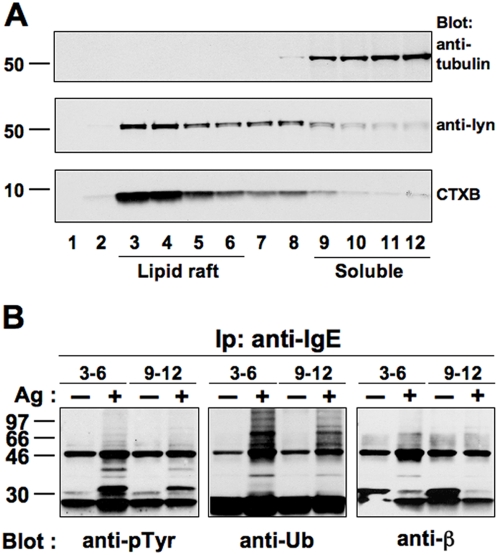
Ubiquitinated forms of FcεRI are found enriched into lipid rafts. (A) RBL-2H3 cells (2×10^7^) were lysed in 0.05% Triton-X100 and fractionated by sucrose density gradient centrifugation. Equal aliquots of the collected fractions were separated by SDS-PAGE, and blotted with anti-tubulin or anti-lyn Abs or revealed using HRP-coniugated CTXB. (B) Fractions identified as raft (3–6) or soluble (9–12) obtained from sensitized RBL-2H3 cells (6×10^7^/sample) stimulated (+) or not (−) with DNP-HSA (Ag) for 1 min at 37°C were pooled, immunoprecipitated with an anti-IgE mAb, separated by SDS-PAGE and immunoblotted with the indicated Abs.

After centrifugation on discontinous sucrose density gradient, 12 fractions were collected and equal volumes of each separated by SDS-PAGE and probed with HRP-conjugated cholera toxin B (CTXB) to label ganglioside GM1 or Abs recognizing tubulin and Lyn (Figure 4A). The lipid raft-associated GM1 was mainly present in the low-density fractions of the gradient (3–6), confirming that these fractions are enriched in lipid rafts. In agreement with previous reports [24], we also found a significant amount of the tyrosine kinase Lyn in raft membranes, whereas tubulin was mainly located in the high-density fractions (9–12).

After having established the validity of our fractionation procedure, equal volume of the fractions enriched in lipid rafts (3–6) and those identified as detergent-soluble (9–12) obtained from unstimulated (−) and stimulated (+) cells, were pooled and immunoprecipitated with an anti-IgE mAb in order to investigate the redistribution of ubiquitinated receptor subunits (Figure 4B). In unstimulated cells, FcεRI β chain was mainly located in the soluble fractions, while upon cross-linking a large percentage of phosphorylated and ubiquitinated receptor subunit was located in the low density fractions. Similar results were obtained for FcεRI γ chain (data not shown).

The ubiquitinated FcεRI β and γ subunits were almost entirely associated with lipid rafts when equivalent amount of proteins were immunoprecipitated from the detergent-insoluble and soluble fractions (data not shown).

Cell pretreatment with UBE1-41 did not abrogate the Ag-induced redistribution of receptor subunits into the detergent-insoluble fractions (Figure S1), supporting the conclusion that the engaged receptor complexes translocate to lipid rafts before the ubiquitination process.

To further investigate whether the integrity of lipid raft is required for receptor ubiquitination, RBL cells were pretreated with 10 mM methyl-β-cyclodextrin (MβCD), a cholesterol depleting agent able to induce the delocalization of aggregated FcεRI and Lyn [26].

**Figure 5:**
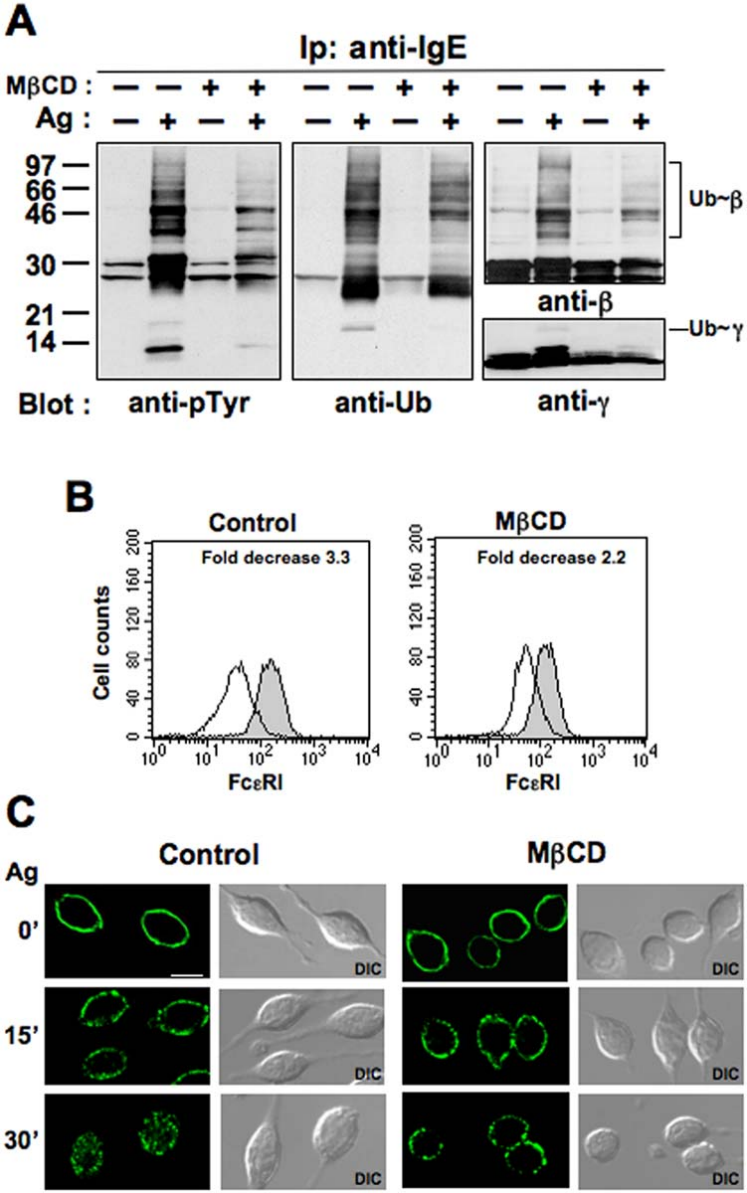
The integrity of lipid rafts is required for FcεRI ubiquitination and internalization. (A) RBL-2H3 cells were pretreated with 10mM MβCD for 30 min at 37°C, a condition able to deplete reproducibly 50% of cellular cholesterol, stimulated (+) or not (−) with Ag for 1 min at 37°C and immunoprecipitated with anti-IgE Ab. The immunoprecipitates were resolved by SDS-PAGE and immunoblotted with the indicated Abs. (B) Cells treated as in *A* were stimulated with Ag for 30 min and the FcεRI surface expression was measured by FACS analysis. Fold decrease was calculated as described in Figure 2B. (C) Cells (120×10^3^) were grown for 12 h on round glass coverslips in the presence of anti-DNP IgE (0.3 µg), treated with 10mM MβCD and stimulated with Ag for the indicated lenghts of time. IgE-FcεRI complexes were visualized with a FITC-conjugated anti-IgE mAb, and fluorescence images, shown as a single optical section, obtained using an ApoTome microscope. The differential interference contrast (DIC) image overlay the fluorescence image is also shown. Scale bar indicates 10 µm. Results are representative of three independent experiments.

A significant reduction of Ag-induced receptor tyrosine phosphorylation was observed upon cholesterol depletion (Figure 5A, left panel), as previously reported [26]. Furthermore, the drug largely prevented FcεRI β and γ ubiquitination (Figure 5A, middle and right panels).

To determine whether FcεRI endocytosis requires intact lipid rafts, we assessed the effect of MβCD pretreatment on the Ag-induced receptor down-modulation and internalization by FACS analysis and fluorescence microscopy. After MβCD pretreatment, a substantial decrease in receptor down-modulation was observed upon 30 min of Ag stimulation (Figure 5B), and correlates with an impairment of receptor internalization (Figure 5C). Upon 15 min of stimulation, small patches of aggregated receptors are formed in both MβCD untreated and treated cells, indicating that lateral mobility is not affected by cholesterol depletion; however, after 30 min of stimulation the engaged receptors mainly remained at the cell surface in the presence of the drug, while the majority of them internalized in control cells.

A complete inhibition of Ag-stimulated lipid raft dependent internalization of FITC-conjugate CTXB bound to GM1 ganglioside was observed upon Ag-stimulation, confirming the efficacy of the drug treatment in inducing raft destruction (Figure S2).

Similar results were obtained by using nystatin, a cholesterol-chelating drug that also interferes with lipid raft functions without extracting sterols from the membrane (data not shown).

All together, these results support a role for specialized membrane domains in controlling FcεRI ubiquitination and internalization.

### UIM-containing adaptors control endocytosis of FcεRI complexes

Likely candidates for coupling mono- or multiubiquitinated receptors to the endocytic machinery are the adaptor proteins harboring Ub interacting motifs (UIMs) Eps15, Eps15R, Epsin1 and Hrs, all of them expressed in RBL cells [34, and data not shown], and involved in the recruitment of ubiquitinated membrane proteins into endocytic compartments [3], [35]–[37].

**Figure 6:**
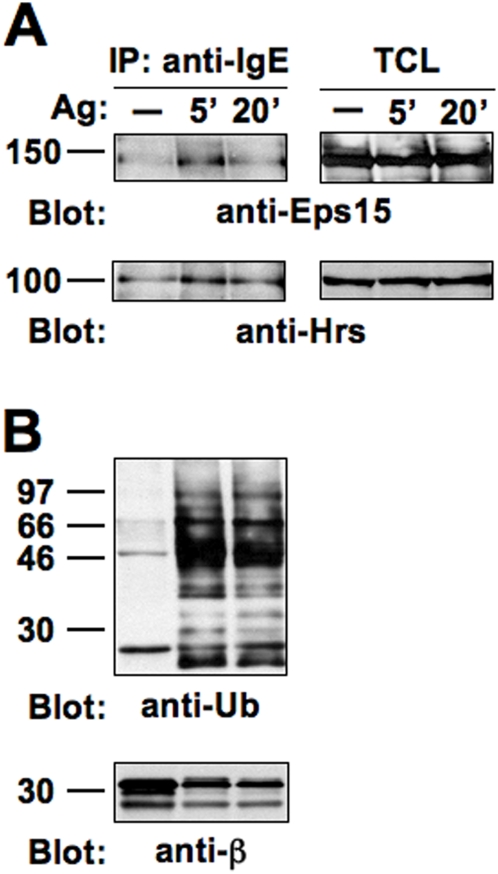
FcεRI interacts with UIM-containing proteins upon stimulation. Sensitized RBL-2H3 cells (6×10^7^) were stimulated with Ag for the indicated lengths of time, lysed and immunoprecipitated with anti-IgE mAb. Immunoprecipitates or total cell lysates (TCL) were resolved by 7.5% (A) or 15% (B) SDS-PAGE and immunoblotted with the indicated Abs.

To investigate whether ubiquitinated FcεRI subunits could interact with UIM-containing proteins, lysates obtained from RBL cells unstimulated (−) and stimulated for the indicated lengths of time were subjected to immunoprecipitation with an anti-IgE mAb, separated by SDS-PAGE, and analyzed by immunoblotting with anti-Eps15 and anti-Hrs Abs (Figure 6A). The relative amount of Eps15 and Hrs associated with FcεRI changed in a time-dependent manner: it was maximal at 5 min after stimulation and decreased after 20 min. The level of FcεRI/UIM protein association remarkably correlated with that of receptor ubiquitination, as shown by the anti-Ub blot (Figure 6B). Immunoblotting with anti-β Ab demonstrated that equivalent amounts of receptor subunits were immunoprecipitated at all time points examined. Eps15R and Epsin1 also co-precipitated with FcεRI complexes (data not shown).

To address the functional role of the UIM-containing proteins in controlling the endocytosis of ubiquitinated FcεRI complexes and their sorting along the endocytic machinery, we used small interfering RNA (siRNA)-based approaches combined with fluorescence microscopy.

Eps15, Eps15R and Epsin1 display similar functions: they control the early steps of the endocytic route coupling ubiquitinated receptors with components of the coated pits [35], [36].

**Figure 7:**
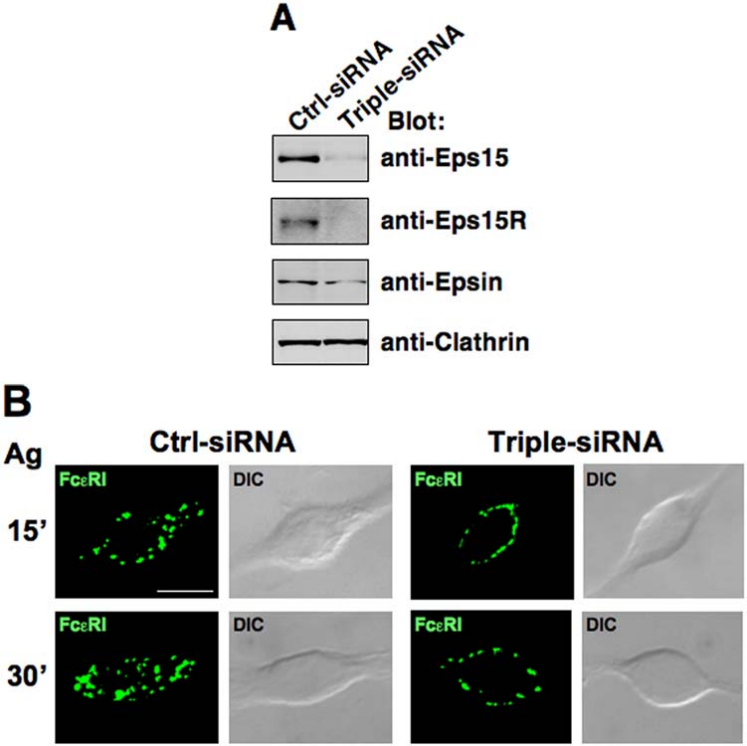
The triple knock-down of Eps15/Eps15R/Epsin1 affects early phases of Ag-induced FcεRI endocytosis. RBL-2H3 cells were simultaneously transfected with Eps15-, Eps15R-, and Epsin1-siRNA or with Ctrl-siRNA. (A) After 24 h total cell lysates were analyzed by Western blotting with the indicated Abs. (B) siRNA transfected cells (120×10^3^) were grown for 12 h on round glass coverslips in the presence of anti-DNP IgE (0.3 µg), and stimulated with Ag for 15 or 30 min as indicated. IgE-FcεRI complexes were visualized with a FITC-conjugated anti-IgE mAb, and fluorescence images, shown as a single optical section, obtained using an ApoTome microscope. Representative Images from over 30 cells examined for each time point are shown. The differential interference contrast (DIC) image overlay the fluorescence image is also shown. Scale bar indicates 10 µm. Results are representative of two independent experiments.

The individual depletion of Eps15, Eps15R or Epsin1 had no effect on receptor endocytosis (data not shown). Thus, we decided to knock-down the three adaptors simultaneously, obtaining a protein level reduction of ∼50% for Epsin1 and a protein depletion of ∼90% for Eps15 and Eps15R when compared with non targeting (Ctrl)-siRNA (Figure 7A).

The triple knock-down did not alter the FcεRI surface expression and distribution on unstimulated cells (data not shown).

Upon 15 min of antigen stimulation at 37°C, microscopic analysis revealed a re-distribution of engaged FcεRI complexes in intracellular dots on control cells and dash like pattern on the surface of triple Eps15/Eps15R/Epsin1 interfered cells (90% of a total of 30 cells examined) (Figure 7B). Notably, after 30 min of stimulation, the majority of engaged receptors were concentrated in intracellular peripheral dots in control cells, while they were still localized in spots on the plasma membrane of the triple knocked-down cells (∼50% of a total of 50 cells examined).

Our results strongly suggest a redundant role for Eps15, Eps15R and Epsin1 in regulating the early steps of Ag-induced FcεRI endocytosis.

**Figure 8:**
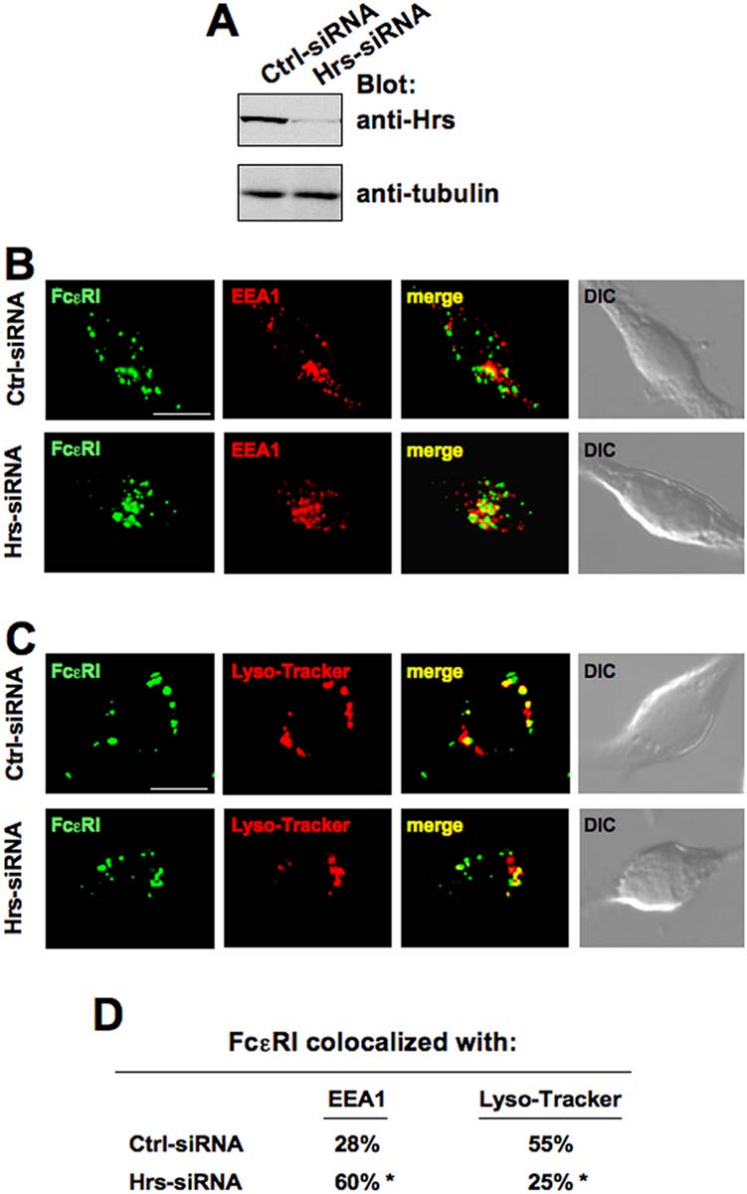
siRNA-mediated knock-down of endogenous Hrs affects Ag-induced FcεRI trafficking. RBL-2H3 cells were transfected with Ctrl- or Hrs-siRNA. (A) After 24 h total cell lysates were analyzed by Western blotting with the indicated Abs. (B and C) Cells were sensitized and stimulated as in Figure 5C for 1 h (B) or 2 h (C), incubated with a FITC-conjugated anti-IgE to visualize IgE-FcεRI complexes (green), stained (red) for EEA1 to identify early endosomes (B) or with Lyso-Tracker (C) to identify late endosomes and lysosomes, and analized with an ApoTome microscope. Red and green images were collected and merged, with yellow coloration indicating colocalization. The differential interference contrast (DIC) image overlay the fluorescence image is also shown. Scale bar indicates 10 µm. Results are representative of three independent experiments. (D) Quantitative analysis of the results shown in B and C. The mean percent of colocalization was calculated analyzing at least 40 cells randomly taken at each time-point from each experiments. Asterisks indicate statistical significance of *p*<0.001 with respect to control.

The key role of Hrs is, instead, the delivery of ubiquitinated proteins to the outer membrane of the late endosomes and the sorting of the cargo into internal vesicles of multivesicular bodies for lysosomal degradation [38], [39]. Thus, we decide to follow and compare the fate of internalized receptor complexes in cells transfected with non targeting siRNA or with Hrs-siRNA. We reproducibly obtained a protein level reduction of ∼80% when compared with Ctrl-siRNA (Figure 8A).

After 1 h of stimulation at 37°C, the colocalization of FcεRI with early endosome Ag 1 (EEA1), a marker of early endosomes, was comparable in control cells while was significantly increased (∼50%) in Hrs depleted cells (Figure 8B; quantification in Figure 8D). The accumulation of FcεRI in early endosomes observed upon Hrs depletion is perfectly compatible with the function of Hrs in regulating the sorting of ubiquitinated proteins from early endosomes to lysosomes for eventual degradation [38], [39].

Notably, Hrs depletion also affected the lysosomal targeting of internalized FcεRI: after 2 h of stimulation, a colocalization of receptor and Lyso-Tracker Red-positive acidic compartments was detected in control cells (Figure 7C), but was partially prevented upon siRNA knock- down of Hrs (55% of colocalization on control cells versus 25% on Hrs-siRNA cells, Figure 8D).

Taken together our results indicate that the triple Eps15/Eps15R/Epsin1-siRNA knock-down negatively affects ligand-induced receptor endocytosis, whereas Hrs-siRNA knock-down causes accumulation of engaged receptors in EEA1-containing vesicles and partially prevents the targeting of activated receptors to a degradative compartment.

## Discussion

Endocytosis of engaged FcεRI complexes appears to be an important regulatory mechanism to ensure a timely limited receptor activity. Although several evidence, including our own, have demonstrated that aggregated FcεRI complexes are mainly endocytosed via clathrin-coated pits [16]–[18], more recent data suggest the existence of an alternative lipid raft-dependent route of receptor internalization [19], [20]. Regardless, the molecular mechanisms responsible for FcεRI entry and trafficking are still largely unknown.

Multiubiquitination of membrane receptors is involved in endocytic trafficking, both at the stage of receptor entry and in the endosomal compartments where ubiquitinated receptors are sorted to a lysosomal compartment for degradation [2], [4]–[6], [8], [9].

We have previously demonstrated a c-Cbl dependent ubiquitination of β and γ FcεRI subunits immediately after receptor engagement [15]. Here, we demonstrate that both receptor subunits are mainly monoubiquitinated at multiple sites upon Ag stimulation (Figure 1 and data not shown).

Under conditions of impaired receptor ubiquitination we reproducibly observed a decrease of FcεRI down-regulation (Figures 2 and 3).

The result could not be explained with an increased recycling, since it has been recently demonstrated on RBL-2H3 cells that aggregated receptor complexes do not recycle back to the cell membrane but are efficiently routed towards late endosomes and lysosomes for degradation [40]. Thus, the decrease of FcεRI down-regulation observed is likely due to a reduced rate of receptor internalization.

Our results are in line with previous studies obtained on membrane proteins in yeast [4], and provide evidence for a role of Ub as an internalization signal for membrane receptors also in mammalian cells.

Additionally, the results obtained using clones lacking active Syk, demonstrate the requirement of Syk kinase activity for efficient Ag-induced FcεRI internalization (Figure 3). Because the presence of active Syk is required for c-Cbl-mediated receptor ubiquitination [15], it is likely that, by controlling receptor ubiquitination, Syk might indirectly affect receptor internalization and trafficking. Another possibility, which is not necessarily mutually exclusive, is that Syk kinase activity might control the action of molecular adaptors other than c-Cbl, directly implicated in the endocytic pathway. We are actually investigating this latter possibility.

We demonstrate that ubiquitinated receptor accumulate into lipid rafts within minutes after their engagement and that receptor ubiquitination is altered by cholesterol depletion (Figures 4 and 5A), arguing in favour of a role for a lipid raft environment in the initiation of receptor ubiquitination. Our results are consistent with recent finding demonstrating that the Ub ligase c-Cbl, responsible for mediating receptor ubiquitination [15], is recruited into lipid rafts upon Ag stimulation in RBL-2H3 cells [30].

Moreover, our finding (Figures 5B and 5C) also suggest an interdependence between lipid rafts and receptor endocytosis, in line with the finding of Fattakhova and coworkers, who demonstrated that aggregated FcεRI complexes remain associated with lipid rafts upon Ag-induced internalization [19].

Thus, a cohesive model is one in which lipid rafts, in addition to assemble the machinery needed for the downstream propagation of the signal from the engaged FcεRI complexes [24]–[28], might also serve as platforms to recruit the necessary machinery for their endocytosis.

Our findings are in line with those of Puri and coworkers who demonstrated that, in the case of EGFR, the lipid rafts are competent for recruitment of both effector signaling molecules and endocytic machinery [41], and those of Stoddart and coworkers who showed a similar situation for clathrin-mediated internalization of BCR [42].

The recruitment of the internalization machinery may include the recently characterizated family of adaptor proteins harboring a UIM domain [43], [44]. Eps15, Eps15R, Epsin1, and Hrs belong to this family of adaptors, bind to ubiquitinated receptors, and colocalize with them in endosomal microdomains [35]–[37].

Despite the lack of enrichment of UIM-containing proteins in purified lipid raft fractions (data not shown), all of them co-immunoprecipitate with endogenous FcεRI in an Ag-dependent fashion and within a time-course compatible with function(s) at different steps of the endocytic route (Figure 6 and data not shown).

Although we failed to observe a significant decrease of FcεRI internalization upon individual depletion of Eps15, Eps15R or Epsin1 (data not shown), the simultaneous depletion of all of them impaired ligand-induced receptor endocytosis (Figure 7B), suggesting a partially overlapping function of these adaptors in ubiquitinated FcεRI uptake. Our results are consistent with previous data showing that only a simultaneous depletion of the three UIM-containing adaptors impairs EGFR endocytosis [45].

Moreover, the requirement of UIM-adaptor proteins for FcεRI endocytosis supports our previous pharmacological evidence that Ag-induced FcεRI ubiquitination might represent a signal for proper receptor internalization.

It should be pointed out that upon 30 min of stimulation 50% of the total population showed an impairment of FcεRI endocytosis (Figure 7B), and upon 1h of stimulation receptors localized in punctate intracellular endocytic dots in all the transfectants analyzed (data not shown).

A likely explanation is that although UIM-adaptors are required for a more rapid and efficient Ag-induced FcεRI endocytosis, redundant mechanisms of receptor endocytosis may exist in RBL-2H3 cells that not necessarily involve Ub and UIM containing proteins. In this respect, we have recently proposed a role for CIN85 and c-Cbl as endocytic adaptors regulating a clathrin-dependent mechanism of FcεRI internalization [18].

The key role of Hrs is the delivery of ubiquitinated proteins to multivesicular bodies for lysosomal degradation [38], [39].

Notably, our results demonstrate a critical role for Hrs in controlling the fate of internalized receptor complexes: Hrs depletion retains ubiquitinated receptors into early endosomes and partially prevents their sorting into lysosomes (Figures 8B and 8C). Our results are consistent with previous studies showing that Hrs depletion induces retention of ubiquitinated EGFR in early endosomes, and impairs degradation of internalized receptors [38], [39].

In summary, our findings shed lights on the central role of lipid raft-dependent FcεRI ubiquitination in controlling the endocytosis of engaged receptors.

We provide the first evidence that Ub might direct Ag-induced endocytosis of immune receptors through interactions with UIM containing proteins. Furthermore, although our data do not resolve whether receptor ubiquitination functions as an internalization signal able to control endocytosis through a clathrin-dependent or independent pathway or a combination of both, they support a key role for the Ub pathway to ensure proper endocytic trafficking of an immune receptor to the lysosomal compartment where degradation of the complexes can take place.

Our findings are compatible with a scenario in which the recruitment of engaged receptor subunits into lipid rafts is required for receptor ubiquitination, and then the ubiquitinated receptors are simply internalized from the plasma membrane location (being raft or not) in which they show competence for interaction with the endocytic machinery including UIM containing proteins.

## Materials and Methods

### Chemical reagents and antibodies

All chemical and drugs, unless otherwise mentioned, were obtained from SIGMA (Sigma-Aldrich, Italy).

The rabbit polyclonal anti-Hrs and anti-EEA1 Abs were a generous gift from Dr. N. Kitamura (Tokyo Institute of Technology, Yokohama, Japan) and Dr. M. Zerial (Max Planck Institute of Molecular Cell Biology and Genetics, Dresden, Germany), respectively; anti-FcεRI β subunit mAb (JRK) was kindly provided by Dr. J.-P. Kinet (Beth Israel Deaconess Medical Center, Boston, MA, USA).

The anti-phosphotyrosine (anti-pTyr) 4G10 mAb was purchased from UBI (Lake Placid, NY). Rabbit anti-Eps15R and anti-Epsin1 polyclonal Abs were from Epitomics (Burlingame, CA, USA). Rabbit anti-Eps15 and anti-Lyn 44 polyclonal Abs and mouse anti-Ub P4D1 and anti-clathrin heavy chain mAbs were purchased from Santa Cruz Biotechnology (Santa Cruz, CA). Monoclonal anti-DNP-specific mouse IgE (clone SPE-7) and anti-β tubulin, HRP-conjugated CTXB, and FITC-conjugated CTXB were purchased from SIGMA.

The mouse anti-Ub FK1 (PW8805) and FK2 (PW8810) Abs were purchased from BIOMOL International, L.P. (Exeter, United Kingdom).

The inhibitor of E1 enzyme, UBE1-41, was from Biogenova (Frederick, Maryland, USA).

Lyso-Tracker Red was purchased from Molecular Probes (Eugene, OR, USA); 4% paraformaldehyde/PBS was from Electron Microscopy Sciences (Washington, PA, USA); Texas Red-conjugated goat anti-rabbit IgG were purchased from Cappel Research Products (Durham, NC, USA).

Purified and FITC-conjugated rat anti-IgE mAbs were purchased from BD Biosciences (San Jose, CA, USA).

### Cell culture and stimulation

RBL-2H3 mast cell line was cultured in monolayers as described previously [15].

The B2 sub-clone derived from a Syk-negative variant of RBL-2H3 cells and cells obtained by stable transfection of the B2 with the WT or a kinase inactive form of Syk [33] have been kindly provided by Drs. J. Zhang and R. P. Siraganian (National Institutes of Health, Bethesda, Maryland, USA).

Adherent cells were incubated with 0.5 µg/ml of monomeric anti-DNP mouse IgE for 12 h at 37°C. The cells were then harvested, resuspended at 10^7^cells/ml in prewarmed EMEM supplemented with 5% FCS, and stimulated by adding 1 µg/ml DNP-HSA for the indicated lengths of time. Stimulation was stopped by addition of cold PBS, and cells lysed as previously described [15]. Lysates were cleared of debris by centrifugation at 15,000×*g* for 20 min; the protein concentration was determined using the Bradford protein assay (Bio-Rad Laboratories, Hercules, CA) with bovine serum albumine (BSA, Amresco) as standard, and the normalized samples were used as total cell lysates or for immunoprecipitation.

To deplete cholesterol, cells (2×10^6^ cells/ml) were incubated in a buffer containing 20 mM Hepes, pH 7.4, 135 mM NaCl, 5 mM KCl, 1.8 mM CaCl_2_, 1 mM MgCl_2_, 5.6 mM glucose, and 1 mg/ml BSA, supplemented or not with 10 mM MβCD for 30 min at 37°C and then stimulated or not in the presence of the same buffer. This treatment reproducible led to ∼50% extraction of cellular cholesterol as revealed by TLC. Under these conditions we did not observe loss of cell surface FcεRI expression.

For experiments requiring inhibition of E1 enzyme, suspended cells (10^7^ cells/ml) were pretreated with 50 µM UBE1-41 or vehicle alone (DMSO) in medium supplemented with 5% FCS for 30 min at 37°C and stimulated or not in the presence of the same medium.

### Sucrose gradient fractionation

Lipid rafts were isolated from RBL-2H3 cells using the method described by B. Baird and D. Holowka [24] with some modifications.

Briefly, sensitized cells were harvested in PBS without divalent cations supplemented with 1.5 mM EDTA, stimulated or not with DNP-HSA, washed two times with ice-cold PBS, and resuspended (25×10^6^ cells/ml) in ice-cold lysis buffer (10 mM Tris-HCl, pH 8, 50 mM NaCl, 10 mM EDTA, 5 mM N-ethyl-maleimide, protease and phosphatase inhibitors) containing 0.05% (v/v) TritonX-100, for 1 h on ice. Lysates were transfered in polypropylene tubes and mixed with an equal volume of 80% sucrose prepared in 25 mM Tris-HCl, pH 7.5, 125 mM NaCl, and 2 mM EDTA. The mixture was overlaid with 2 ml of 30% sucrose and 1 ml of 5% sucrose. After centrifugation at 48000 rpm for 18 h at 4°C using a swing SW60 rotor (Beckman Instruments Inc., Palo Alto, CA, USA), 12 fractions of 0.4 ml each were harvested from the top of the gradient and equal volumes of each resuspended in SDS buffer, separated by SDS-PAGE and analyzed in Western blotting.

The purity of detergent-insoluble fractions was verified by probing with HRP-conjugated CTXB to localize GM1 and with rabbit anti-Lyn Ab, and the purity of detergent-soluble fractions by probing with anti-β tubulin mAb.

In some experiments, the detergent insoluble- and soluble-fractions were pooled and diluted with lysis buffer containing a final concentration of 0.5% (v/v) TritonX-100 for subsequent FcεRI immunoprecipitation.

### Immunoprecipitation, electrophoresis and immunoblotting

For immunoprecipitation, postnuclear supernatants were precleared by mixing with protein G-Sepharose beads for 1 h at 4°C, and immunoprecipitated with the indicated Abs pre-bound to protein G beads. After gentle rotation at 4°C for 2–12 h the beads were washed five times with lysis buffer and bound proteins eluted with Laemmli buffer (75 mM Tris-HCl, pH 6.8, 2% SDS, 10% glycerol, and 1% 2-βME), resolved by SDS-PAGE and transferred electrophoretically to nitrocellulose filters. After blocking nonspecific reactivity, filters were probed with specific Abs diluted in TBS-T (20 mM Tris-HCl pH 8, 150 mM NaCl, and 0.05% Tween 20). After extensive washing in TBS-T, the membranes were incubated with HRP-labeled GAM Ig or goat anti-rabbit Ig Abs (Amersham Biosciences, Italy) and immunoreactivity was visualized by using the ECL system (Amersham Biosciences, Italy). Densitometric analysis of the films was performed using the NIH Image J software.

### FcεRI down-modulation assay

The cells were sentitized with anti-DNP mouse IgE as above described, harvested, and 5×10^5^ cells/sample were resuspended in 50 µl of serum-free medium added or not with 1 µg/ml DNP DNP-HSA and incubated for 30 min at 37°C to induce receptor internalization. Control samples were kept on ice for the same time points. Endocytosis was stopped by addition of 0.1% NaN_3_ in cold PBS for 5 min. In order to evaluate FcεRI surface expression, samples were labelled with FITC-conjugated anti-mouse IgE and the cytofluorimetric analysis was performed with a FACSCalibur flow cytometer (Becton Dickinson Immunocytometry Systems).

### Small interfering RNA (siRNA)

Eps15-siRNA (5′-CUUUAUUGCUUUACGGCUU-3′), Eps15R-siRNA (5′-ACAGAAAGCCAGCGAAGUATT-3′), Epsin1-siRNA (5′-ACGAUAAGGAAGAGCGGAUTT-3′), Hrs-siRNA (5′-CAAGAUACCUCAACCGGAA-3′), and a control non targeting siRNA (5′-UAAGGCUAUGAAGAGAUACUUTT-3′) were purchased from Eurofins MWG Operon (Ebersberg, Germany). siRNA duplexes (100 µM) were resuspended in 1X siRNA Universal Buffer. Specific protein knock-down was achieved by transfecting RBL-2H3 cells with siRNA duplexes. The transfection was performed by electroporation (310 V, 960 µF) incubating 1×10^7^ cells with 2.5 µM siRNA in 500 µl of serum-free MEM.

For triple transfections with Eps15-, Eps15R- and Epsin1-siRNA duplexes, the amount of each duplex was reduced in order to maintain the final siRNA concentration at 2.5 µM, and a second transfection was repeated after 24 h.

After 24 h from the last transfection, cells were processed for Western blotting and microscopic analysis.

### Immunofluorescence and microscopic analysis

Cells were grown for 24 h in MEM supplemented with 16% FCS on round glass coverslips coated with 2% gelatin (120×10^3^/well) and incubated with anti-DNP IgE (0.3 µg/well) overnight. After extensive washing in cold medium, cells were kept on ice or stimulated with 0.5 µg/ml DNP-HSA for the indicated lengths of time to induce receptor internalization. Stimulation was blocked washing with cold PBS, and fixing for 30 min at room temperature in 4% paraformaldehyde/PBS. The fixed cells were then incubated for 20 min at room temperature with 0.1 M glicine/PBS and permeabilized for 5 min in PBS/0.1% saponin/5% FCS/2% BSA. Coverslips were rinsed with PBS and incubated for 30 min with FITC-conjugated anti-IgE.

To visualize early endosomes, after the labelling with anti-IgE FITC, cells were extensively washed with PBS and incubated for 1 h at room temperature with rabbit polyclonal anti-EEA1, washed and incubated for 30 min at room temperature with Texas Red-conjugated goat anti-rabbit Ig diluted in PBS /0.1% saponin/5% FCS/2% BSA.

To identify late endosomes and lysosomes cells were incubated with 300 nM Lyso-Tracker Red at 37°C for the last 30 min during stimulation.

Images were acquired using the Apotome Zeiss microscope Observer Z.1 (Carl Zeiss, Jena, Germany) equipped with a ×40 objective. Apotome Zeiss system provides an optical slice view reconstructed from fluorescent samples, using a series of “grid projection” acquisitions.

Colocalization of the fluorescence signals was analyzed with AxioVision 4.6.3 software.

The mean percent of colocalization was calculated analyzing a minimum of 40 cells randomly taken for each experiments and *p* values were calculated using Student's *t* test.

## Supporting Information

Figure S1 The impairment of FcεRI ubiquitination does not affect translocation of engaged receptors into lipid rafts. (A) Sensitized RBL-2H3 cells were pretreated for 30 min with 50 µM UBE1-41, stimulated with Ag for the indicated lengths of time, lysed in 0.05% Triton-X100 and fractionated by sucrose density gradient centrifugation. Fractions identified as raft (3–6) or soluble (9–12) obtained from unstimulated (−) and Ag stimulated (+) cells, were pooled, separated by SDS-PAGE and immunoblotted with the indicated Abs. (B) Pooled fractions obtained as in A were immunoprecipitated with anti-IgE, separated by SDS-PAGE and immunoblotted with anti-β mAb.
(2.19 MB TIF)

Figure S2 MβCD inhibits FcεRI-induced CTXB internalization. Cells (120×10^3^) were grown for 12 h on round glass coverslips in the presence of anti-DNP IgE (0.3 µg), treated for 30 min with 10 mM MβCD at 37°C, and stimulated with Ag for the indicated lenghts of time. GM1 was visualized with a FITC-conjugated CTXB, and fluorescence images, shown as a single optical section, obtained using an ApoTome microscope. The differential interference contrast (DIC) image overlay the fluorescence image is also shown. Scale bar indicates 10 µm. Results are representative of three independent experiments.
(3.08 MB TIF)

## References

[1] Ciechanover A, Orian A, Schwartz AL (2000). The ubiquitin-mediated proteolytic pathway: mode of action and clinical implications.. J Cell Biochem Suppl.

[2] Hicke L, Dunn R (2003). Regulation of membrane protein transport by ubiquitin and ubiquitin-binding proteins.. Annu Rev Cell Dev Biol.

[3] Mukhopadhyay D, Riezman H (2007). Proteasome-independent functions of ubiquitin in endocytosis and signaling.. Science.

[4] Shih SC, Sloper-Mould KE, Hicke L (2000). Monoubiquitin carries a novel internalization signal that is appended to activated receptors.. EMBO J.

[5] Longva KE, Blystad FD, Stang E, Larsen AM, Johannessen LE (2002). Ubiquitination and proteasomal activity is required for transport of the EGF receptor to inner membranes of multivesicular bodies.. J Cell Biol.

[6] Haglund K, Sigismund S, Polo S, Szymkiewicz I, Di Fiore PP (2003). Multiple monoubiquitination of RTKs is sufficient for their endocytosis and degradation.. Nat Cell Biol.

[7] Jiang X, Sorkin A (2003). Epidermal growth factor receptor internalization through clathrin-coated pits requires Cbl RING finger and proline-rich domains but not receptor polyubiquitylation.. Traffic.

[8] Duan L, Miura Y, Dimri M, Majumder B, Dodge IL (2003). Cbl-mediated ubiquitinylation is required for lysosomal sorting of epidermal growth factor receptor but is dispensable for endocytosis.. J Biol Chem.

[9] Stang E, Blystad FD, Kazazic M, Bertelsen V, Brodahl T (2004). Cbl-dependent ubiquitination is required for progression of EGF receptors into clathrin-coated pits.. Mol Biol Cell.

[10] Siraganian RP (2003). Mast cell signal transduction from the high-affinity IgE receptor.. Curr Opin Immunol.

[11] Yamasaki S, Saito T (2005). Regulation of mast cell activation through FcεRI.. Chem Immunol Allergy.

[12] Kraft S, Kinet JP (2007). New developments in FcεRI regulation, function and inhibition.. Nat Rev Immunol.

[13] Rivera J, Fierro NA, Olivera A, Suzuki R (2008). New insights on mast cell activation via the high affinity receptor for IgE.. Adv Immunol.

[14] Paolini R, Kinet JP (1993). Cell surface control of the multiubiquitination and deubiquitination of high-affinity immunoglobulin E receptors.. EMBO J.

[15] Paolini R, Molfetta R, Beitz LO, Zhang J, Scharenberg AM (2002). Activation of Syk tyrosine kinase is required for c-Cbl-mediated ubiquitination of FcεRI and Syk in RBL cells.. J Biol Chem.

[16] Santini F, Keen JH (1996). Endocytosis of activated receptors and clathrin-coated pit formation: deciphering the chicken or egg relationship.. J Cell Biol.

[17] Wilson BS, Pfeiffer JR, Oliver JM (2000). Observing FcεRI signaling from the inside of the mast cell membrane.. J Cell Biol.

[18] Molfetta R, Belleudi F, Peruzzi G, Morrone S, Leone L (2005). CIN85 regulates the ligand-dependent endocytosis of the IgE receptor: a new molecular mechanism to dampen mast cell function.. J Immunol.

[19] Fattakhova G, Masilamani M, Borrego F, Gilfillan AM, Metcalfe DD (2006). The high-affinity immunoglobulin-E receptor (FcεRI) is endocytosed by an AP-2/clathrin-independent, dynamin-dependent mechanism.. Traffic.

[20] Oliver C, Fujimura A, Silveira E Souza AM, Orlandini de Castro R (2007). Mast cell-specific gangliosides and FcεRI follow the same endocytic pathway from lipid rafts in RBL-2H3 cells.. J Histochem Cytochem.

[21] Simons K, Toomre D (2000). Lipid rafts and signal transduction.. Nat Rev Mol Cell Biol.

[22] Kusumi A, Koyama-Honda I, Suzuki K (2004). Molecular dynamics and interactions for creation of stimulation-induced stabilized rafts from small unstable steady-state rafts.. Traffic.

[23] Mayor S, Rao M (2004). Rafts: scale-dependent, active lipid organization at the cell surface.. Traffic.

[24] Field KA, Holowka D, Baird B (1995). FcεRI-mediated recruitment of p53/56lyn to detergent-resistant membrane domains accompanies cellular signaling.. Proc Natl Acad Sci USA.

[25] Field KA, Holowka D, Baird B (1997). Compartmentalized activation of the high affinity immunoglobulin E receptor within membrane domains.. J Biol Chem.

[26] Sheets ED, Holowka D, Baird B (1999). Critical role for cholesterol in Lyn-mediated tyrosine phosphorylation of FcεRI and their association with detergent-resistant membranes.. J Cell Biol.

[27] Young RM, Holowka D, Baird B (2003). A lipid raft environment enhances Lyn kinase activity by protecting the active site tyrosine from dephosphorylation.. J Biol Chem.

[28] Wilson BS, Steinberg SL, Liederman K, Pfeiffer JR, Surviladze Z (2004). Markers for detergent-resistant lipid rafts occupy distinct and dynamic domains in native membranes.. Mol Biol Cell.

[29] Kovarova M, Wassif CA, Odom S, Liao K, Porter FD (2006). Cholesterol deficiency in a mouse model of Smith-Lemli-Opitz syndrome reveals increased mast cell responsiveness.. J Exp Med.

[30] Lafont F, Simons K (2001). Raft-partitioning of the ubiquitin ligases Cbl and Nedd4 upon IgE-triggered cell signaling.. Proc Natl Acad Sci USA.

[31] Fujimuro M, Yokosawa H (2005). Production of antipolyubiquitin monoclonal antibodies and their use for characterization and isolation of polyubiquitinated proteins.. Methods Enzymol.

[32] Yang Y, Kitagaki J, Dai RM, Tsai YC, Lorick KL (2007). Inhibitors of ubiquitin-activating enzyme (E1), a new class of potential cancer therapeutics.. Cancer Res.

[33] Zhang J, Berenstein EH, Evans RL, Siraganian RP (1996). Transfection of Syk protein tyrosine kinase reconstitutes high affinity IgE receptor-mediated degranulation in a Syk-negative variant of rat basophilic leucemia RBL-2H3 cells.. J Exp Med.

[34] Murai S, Kitamura N (2000). Involvement of hrs binding protein in IgE receptor-triggered exocytosis in RBL-2H3 mast cells.. Biochem Biophys Res Commun.

[35] Carbone R, Fré S, Iannolo G, Belleudi F, Mancini P (1997). eps15 and eps15R are essential components of the endocytic pathway.. Cancer Res.

[36] Chen H, Fre S, Slepnev VI, Capua MR, Takei K (1998). Epsin is an EH-domain-binding protein implicated in clathrin-mediated endocytosis.. Nature.

[37] Raiborg C, Bache KG, Gillooly DJ, Madshus IH, Stang E (2002). Hrs sorts ubiquitinated proteins into clathrin-coated microdomains of early endosomes.. Nat Cell Biol.

[38] Lu Q, Hope LW, Brasch M, Reinhard C, Cohen SN (2003). TSG101 interaction with HRS mediates endosomal trafficking and receptor down-regulation.. Proc Natl Acad Sci USA.

[39] Bache KG, Brech A, Mehlum A, Stenmark H (2003). Hrs regulates multivesicular body formation via ESCRT recruitment to endosomes.. J Cell Biol.

[40] Fattakhova GV, Masilamani M, Narayanan S, Borrego F, Gilfillan AM (2009). Endosomal trafficking of the ligated FcεRI receptor.. Mol Immunol.

[41] Puri C, Tosoni D, Comai R, Rabellino A, Segat D (2005). Relationships between EGFR signaling-competent and endocytosis-competent membrane microdomains.. Mol Biol Cell.

[42] Stoddart A, Jackson AP, Brodsky FM (2005). Plasticity of B cell receptor internalization upon conditional depletion of clathrin.. Mol Biol Cell.

[43] Hofmann K, Falquet L (2001). A ubiquitin-interacting motif conserved in components of the proteasomal and lysosomal protein degradation systems.. Trends Biochem Sci.

[44] Polo S, Sigismund S, Faretta M, Guidi M, Capua MR (2002). A single motif responsible for ubiquitin recognition and monoubiquitination in endocytic proteins.. Nature.

[45] Sigismund S, Woelk T, Puri C, Maspero E, Tacchetti C (2005). Clathrin-independent endocytosis of ubiquitinated cargos.. Proc Natl Acad Sci U S A.

