# Supplementary figures and images for: Lipid Raft-Dependent FcεRI Ubiquitination Regulates Receptor Endocytosis through the Action of Ubiquitin Binding Adaptors
Source: PLoS One. 2009 May 19;4(5):e5604. doi: 10.1371/journal.pone.0005604 (PMC2680016; doi:10.1371/journal.pone.0005604)

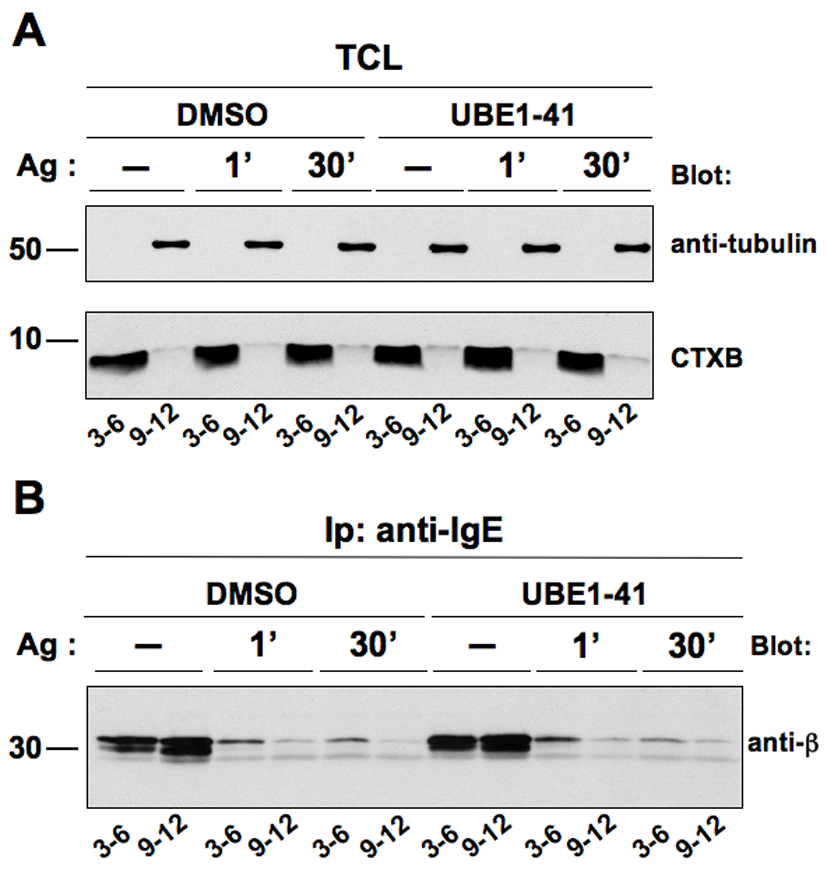

Supplement: Figure S1 — The impairment of FcεRI ubiquitination does not affect translocation of engaged receptors into lipid rafts. (A) Sensitized RBL-2H3 cells were pretreated for 30 min with 50 µM UBE1-41, stimulated with Ag for the indicated lengths of time, lysed in 0.05% Triton-X100 and fractionated by sucrose density gradient centrifugation. Fractions identified as raft (3–6) or soluble (9–12) obtained from unstimulated (−) and Ag stimulated (+) cells, were pooled, separated by SDS-PAGE and immunoblotted with the indicated Abs. (B) Pooled fractions obtained as in A were immunoprecipitated with anti-IgE, separated by SDS-PAGE and immunoblotted with anti-β mAb. (2.19 MB TIF) [file pone.0005604.s001.tif]

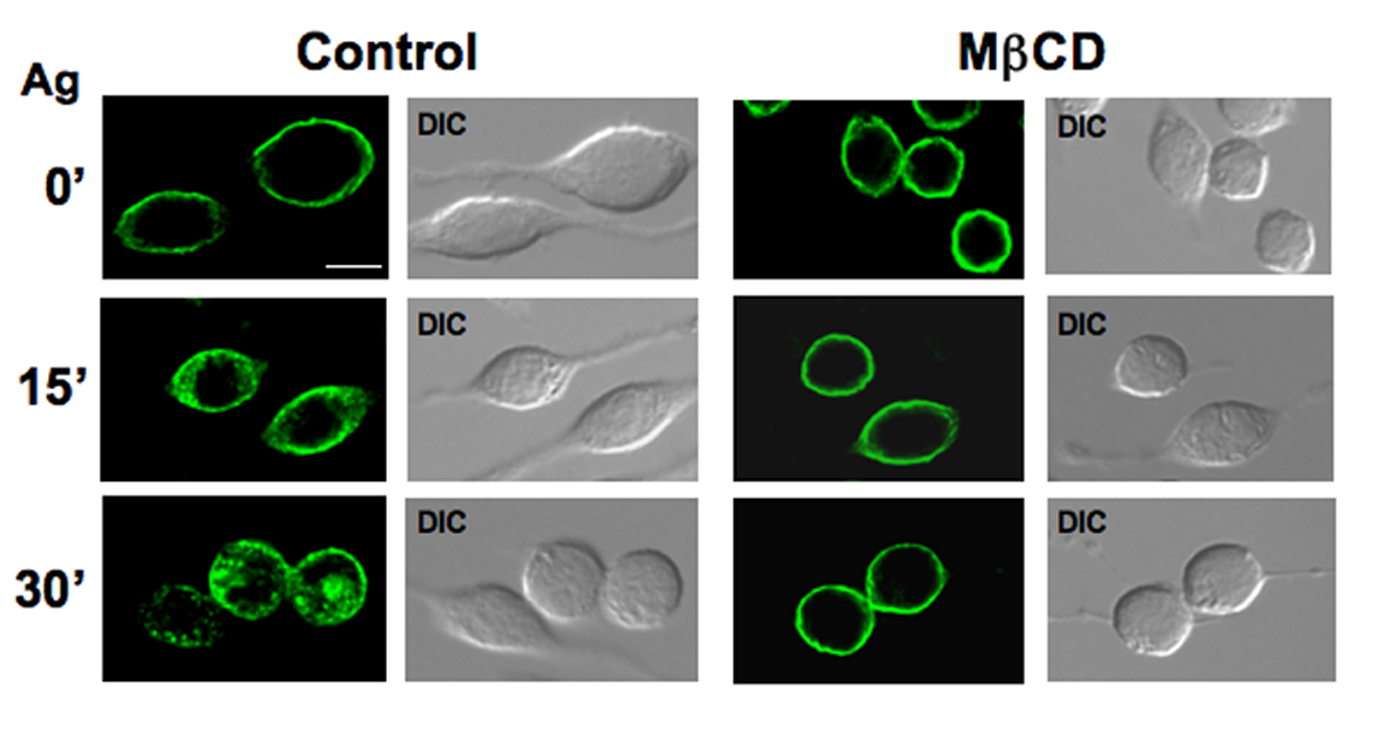

Supplement: Figure S2 — MβCD inhibits FcεRI-induced CTXB internalization. Cells (120×103) were grown for 12 h on round glass coverslips in the presence of anti-DNP IgE (0.3 µg), treated for 30 min with 10 mM MβCD at 37°C, and stimulated with Ag for the indicated lenghts of time. GM1 was visualized with a FITC-conjugated CTXB, and fluorescence images, shown as a single optical section, obtained using an ApoTome microscope. The differential interference contrast (DIC) image overlay the fluorescence image is also shown. Scale bar indicates 10 µm. Results are representative of three independent experiments. (3.08 MB TIF) [file pone.0005604.s002.tif]
